# Reduction and Morphological Transformation of Synthetic Nanophase Iron Oxide Minerals by Hyperthermophilic Archaea

**DOI:** 10.3389/fmicb.2018.01550

**Published:** 2018-07-11

**Authors:** Srishti Kashyap, Elizabeth C. Sklute, M. Darby Dyar, James F. Holden

**Affiliations:** ^1^Department of Microbiology, University of Massachusetts, Amherst, MA, United States; ^2^Department of Astronomy, Mount Holyoke College, South Hadley, MA, United States; ^3^Planetary Science Institute, Tucson, AZ, United States

**Keywords:** hyperthermophile, archaea, iron oxide, magnetite, nanophase

## Abstract

Fe(III) (oxyhydr)oxides are electron acceptors for some hyperthermophilic archaea in mildly reducing geothermal environments. However, the kinds of iron oxides that can be used, growth rates, extent of iron reduction, and the morphological changes that occur to minerals are poorly understood. The hyperthermophilic iron-reducing crenarchaea *Pyrodictium delaneyi* and *Pyrobaculum islandicum* were grown separately on six different synthetic nanophase Fe(III) (oxyhydr)oxides. For both organisms, growth on ferrihydrite produced the highest growth rates and the largest amounts of Fe(II), although *P. delaneyi* produced four times more Fe(II) (25 mM) than *P. islandicum* (6 mM). Both organisms grew on lepidocrocite and akaganéite and produced 2 and 3 mM Fe(II). Modest growth occurred for both organisms on goethite, hematite, and maghemite where ≤1 mM Fe(II) was produced. The diameters of the spherical mineral end-products following *P. delaneyi* growth increased by 30 nm for ferrihydrite and 50–150 nm for lepidocrocite relative to heated abiotic controls. For akaganéite, spherical particle sizes were the same for *P. delaneyi*-reacted samples and heated abiotic controls, but the spherical particles were more numerous in the *P. delaneyi* samples. For *P. islandicum*, there was no increase in grain size for the mineral end-products following growth on ferrihydrite, lepidocrocite, or akaganéite relative to the heated abiotic controls. High-resolution transmission electron microscopy of lattice fringes and selected-area electron diffraction of the minerals produced by both organisms when grown on ferrihydrite showed that magnetite and/or possibly maghemite were the end-products while the heated abiotic controls only contained ferrihydrite. These results expand the current view of bioavailable Fe(III) (oxyhydr)oxides for reduction by hyperthermophilic archaea when presented as synthetic nanophase minerals. They show that growth and reduction rates are inversely correlated with the iron (oxyhydr)oxide crystallinity and that iron (oxyhydr)oxide mineral transformation takes different forms for these two organisms.

## Introduction

Microbes and minerals have been intimately associated with each other throughout Earth’s history (Hazen et al., 2008). Examining their interactions, transformations, and co-evolution is essential to understanding early life on Earth as well as its potential existence on other rocky worlds. One microbial process that is relevant in this regard is dissimilatory iron reduction. At near-neutral pH, insoluble Fe(III) (oxyhydr)oxide minerals are the most environmentally relevant and thermodynamically favorable forms of iron for microbial respiration (Straub et al., 2001). These include ferrihydrite (5Fe_2_O_3_∙9H_2_O), lepidocrocite (γ-FeOOH), akaganéite (β-FeOOH), goethite (α-FeOOH), hematite (α-Fe_2_O_3_), and maghemite (γ-Fe_2_O_3_) with varying crystallinities and thermodynamic stabilities (Cornell and Schwertmann, 2003). While ferrihydrite is the predominant form of iron in anaerobic non-sulfidogenic environments (Lovley, 1991), other more crystalline Fe(III) (oxyhydr)oxides can be more abundant in other environments such as soils, shallow aquifers, sediments, and the subsurface (Cornell and Schwertmann, 2003). More recently, a range of Fe(III) (oxyhydr)oxides has been identified in geothermal environments, including sulfide chimneys and microbial mats at various deep-sea hydrothermal vents (Kristall et al., 2006; Toner et al., 2012, 2016; Lin et al., 2014, 2016b), and acidic terrestrial hot springs (Kozubal et al., 2012). In sulfide chimneys and seafloor sulfide deposits at hydrothermal vents, two-line ferrihydrite, six-line ferrihydrite, lepidocrocite, akaganéite, goethite, and a biogenic Fe(III) (oxyhydr)oxide have been identified (Lin et al., 2016b; Toner et al., 2016). Several factors can affect the bioavailability and reactivity of Fe(III) (oxyhydr)oxides including crystallinity and crystallite morphology (Cutting et al., 2009), surface area (Roden and Zachara, 1996; Roden, 2003, 2006), solubility (Bonneville et al., 2004, 2009), size, and degree of aggregation (Yan et al., 2008; Bose et al., 2009; Cutting et al., 2009; Bosch et al., 2010). Abiotic dissolution rates of these minerals using ascorbic acid decrease in the order ferrihydrite > lepidocrocite > goethite > hematite (Postma, 1993). This suggests that less thermodynamically stable, often referred to as less crystalline, Fe(III) (oxyhydr)oxides are more reactive. Yet, because type, crystallinity, size, surface area, and solubility are not mutually exclusive, our understanding of the mineralogical constraints on the rate and extent of iron reduction is incomplete.

Most microbial iron reduction studies have focused on Fe(III) (oxyhydr)oxide reduction by mesophilic *Proteobacteria*, namely *Geobacter* and *Shewanella* species (Shi et al., 2016). *Geobacter* and *Shewanella* reduce ferrihydrite either to superparamagnetic (SPM) magnetite (Fe_3_O_4_) crystals with crystal sizes less than 30 nm and irregular morphologies (Lovley et al., 1987; Sparks et al., 1990; Hanzlik et al., 1996; Zachara et al., 2002; Roh et al., 2006; Li et al., 2009) or to single-domain (SD) magnetite crystals with sizes of 30–120 nm and varied tabular platelet, lath-like, and diamond morphologies (Vali et al., 2004; Perez-Gonzalez et al., 2010). Growth and X-ray diffraction (XRD) studies showed that *Geobacter sulfurreducens* transformed lepidocrocite and akaganéite into mixtures of magnetite and goethite with the proportion of magnetite increasing over time. Growth on hematite and goethite showed limited iron reduction (Cutting et al., 2009). Thus, for *G. sulfurreducens*, less thermodynamically stable Fe(III) (oxyhydr)oxides are more bioreactive than the more thermodynamically stable phases. *G. sulfurreducens* also reduced colloidal ferrihydrite, hematite, goethite, and akaganéite up to 100-fold faster than bulk macroaggregates of the same iron phases (Bosch et al., 2010). This suggests that the reactivity of Fe(III) (oxyhydr)oxides increases with decreasing mineral size regardless of the mineral type due to an increase in mineral surface area. This has biogeochemical relevance because most natural Fe(III) (oxyhydr)oxides exist at sizes of only tens of nanometers in one or more crystallographic direction (Braunschweig et al., 2013). Therefore, mineral type, size, and morphology of Fe(III) (oxyhydr)oxides impact their reactivity.

Dissimilatory iron reduction is also catalyzed by hyperthermophilic archaea in geothermal environments such as hydrothermal vents and hot springs. For example, *Pyrodictium delaneyi* and *Pyrobaculum islandicum* reduce ferrihydrite to magnetite during growth (Kashefi and Lovley, 2000; Kashefi et al., 2008; Lin et al., 2014). *P. islandicum* also has been shown to grow poorly on uncharacterized goethite and hematite (Kashefi and Lovley, 2000). *P. delaneyi* was unable to grow on macroparticulate lepidocrocite, hematite, maghemite, and goethite (Lin et al., 2014). Except for ferrihydrite, “nanophase” (1–100 nm in at least one direction) Fe(III) (oxyhydr)oxides have not been tested for hyperthermophilic iron reduction. Given their increased reactivity relative to bulk phases, they represent an unexplored area for culturing and enrichment among hyperthermophilic archaea. The purpose of this study was to screen *P. delaneyi* and *P. islandicum* for their ability to grow on synthetic nanophase ferrihydrite, lepidocrocite, akaganéite, goethite, hematite, and maghemite. The morphological transformations of ferrihydrite, lepidocrocite, and akaganéite (the three most bioavailable minerals) by *P. delaneyi* and *P. islandicum* were then characterized using transmission electron microscopy (TEM). Ferrihydrite mineral transformation was further studied using high-resolution TEM coupled with selected-area electron diffraction (SAED) analyses. The goal of this work is to understand the mineralogical constraints on Fe(III) (oxyhydr)oxide reduction by hyperthermophilic archaea and the types of mineral morphological transformations that occur.

## Materials and Methods

### Organisms Used

*Pyrodictium delaneyi* Su06^T^ (DSM 28599) and *Pyrobaculum islandicum* GEO3^T^ (DSM 4184) were provided by the Deutsche Sammlung von Mikroorganismen und Zellkulturen. *P. delaneyi* is a marine hyperthermophile isolated from a hydrothermal “black smoker” chimney (Lin et al., 2014, 2016a). *P. islandicum* is a terrestrial hyperthermophile isolated from a solfataric geothermal pool (Huber et al., 1987).

### Mineral Syntheses

Ten nanophase Fe(III) (oxyhydr)oxides were synthesized for this study: two-line ferrihydrite (Fh), akaganéite (Ak102315), goethite (Goet011515 and Goet012315), lepidocrocite (Lep030415 and Lep100615), hematite (Hem100915 and Hem022015), maghemite (Magh061815), and magnetite (Mag060516) (Supplementary Figure S1). The synthesis procedures for these minerals, except Lep100615 and Hem022015, were described previously (Sklute et al., 2018). Hem022015 was synthesized based on hematite “Method 5” and Lep100615 following the main synthesis method in Schwertmann and Cornell (2000). All mineral syntheses were confirmed for phase purity and identity using TEM; visible and near infrared, mid-infrared, and Raman spectroscopies (Sklute et al., 2018); powder XRD; and Mössbauer spectroscopy. All the synthetic Fe(III) (oxyhydr)oxides were kept in solution at 4°C to maintain fluid-mineral properties and prevent drying induced changes in phase and crystallinity.

### Growth and Experimental Conditions

*Pyrodictium delaneyi* was grown in modified DSM 1210 medium as previously described (Lin et al., 2014). The headspace was flushed and pressurized with 200 kPa of H_2_-CO_2_ (80–20%), which also served as the primary carbon and energy sources. *P. islandicum* was grown in medium containing 0.5% (wt vol^-1^) casein hydrolysate as previously described (Feinberg et al., 2008). The terminal electron acceptors for both organisms in separate incubations were 100 mmol l^-1^ each of synthetic nanophase ferrihydrite (Fh), lepidocrocite (Lep030415 and Lep100615), akaganéite (Ak102315), maghemite (Magh061815), goethite (Goet011515 and Goet012315), and hematite (Hem100915 and Hem022015). Growth screening and kinetic experiments were carried out in serum bottles sealed with butyl rubber stoppers. Prior to inoculation, all media were reduced with 0.5 mM cysteine-HCl to remove any residual O_2_ and supplemented with 1.3 mM FeCl_2_∙2H_2_O to initiate reduction. Each bottle was inoculated with a logarithmic growth-phase culture that had been grown and transferred at least three times on the electron acceptor of interest. Each experiment included duplicate inoculated bottles (heat+cells) and an uninoculated control bottle (heat only) to account for any abiotic iron reduction. *P. delaneyi* and *P. islandicum* were incubated in a force air-incubator at 90 and 95°C, respectively. At various time points, the bottles were mixed by hand and an aliquot from each bottle was removed to determine cell and Fe(II) concentrations.

For cell counts, samples were preserved using 2% (vol vol^-1^) formaldehyde and mixed with oxalate solution (28 g l^-1^ ammonium oxalate and 15 g l^-1^ oxalic acid) to dissolve the particulate iron. Cells were filtered onto 0.2 μm pore size black polycarbonate membrane filters (Sterlitech Corporation, Kent, WA, United States), stained with 0.1% (wt. vol^-1^) acridine orange for 3 min, and counted using epifluorescence microscopy (Hobbie et al., 1977). Specific growth rate (*k*) was determined using a best-fit exponential curve fitted to the logarithmic portion of the growth data. Fe(II) concentrations were measured spectrophotometrically using the ferrozine assay (Phillips and Lovley, 1987) and corrected using the results from heated abiotic controls. Fe(II) production yield (*Y*) during growth was determined by plotting Fe(II) concentration against cell concentration for the logarithmic portion of the data, assuming a linear relationship between the two during growth. Cell specific Fe(II) production rates were calculated from (*Y* × *k)*/0.693 as previously described (Lin et al., 2014). Statistical significance for growth, Fe(II) yield, and cell-specific Fe(II) production rates were calculated using linear regression analysis and reported as 95% confidence intervals.

Additional control experiments were run to determine if growth and Fe(II) production by *P. delaneyi* and *P. islandicum* on the various Fe(III) (oxyhydr)oxides were a result of dissimilatory iron reduction or a fortuitous secondary process. Variations tested included (1) uninoculated and (2) inoculated media containing no terminal electron acceptor, (3) only 1.3 mM FeCl_2_∙2H_2_O, and (4) only 1.3 mM FeCl_3_∙6H_2_O, each of which was incubated in the appropriate growth medium and incubation temperature for both organisms. The ferric chloride control incubation was included to account for any potential oxidation of the supplemental 1.3 mM FeCl_2_∙H_2_O that could then be used as a terminal electron acceptor. Furthermore, a dead cell control experiment was designed using a heat-killed late-logarithmic phase culture as the inoculum. For that experiment, a cell pellet of *P. delaneyi* grown on 10 mM KNO_3_ or *P. islandicum* grown on 20 mM Na_2_S_2_O_3_ was obtained by centrifugation at 10,000 × g. Each pellet was re-suspended in 1 ml of the base medium without iron and autoclaved at 121°C for 1 h. The heat-killed re-suspended pellet was then used as the inoculum. These samples were incubated for up to 64 h in duplicate to test for abiotic iron reduction.

### Transmission Electron Microscopy and Selected-Area Electron Diffraction

To examine the mineral end products for their morphology and diffraction patterns, 1 ml of a late logarithmic growth phase culture and its associated “heat only” control was placed into a sealed HPLC vial flushed with N_2_ in an anoxic chamber. Once settled, the mineral was agitated using a pipette tip and 6 μl of sample was applied directly to a 400 mesh copper grid coated with 5–6 nm of formvar and stabilized with a 3–4 nm of carbon film. The sample was left on the grid for 1 min, then excess liquid was gently removed from the corner of the grid using filter paper. To minimize oxidation of the mineral products, all grids were prepared immediately prior to analysis. Grids were first visualized at Mount Holyoke College using a Phillips CM100 transmission electron microscope operated at 100 kV and equipped with a single-tilt goniometer stage and an AMT digital camera. Images were captured using the digital camera and pixel size was calibrated using a catalase standard. This set of electron micrographs was used to assess morphological mineral transformation and variation with organism and electron acceptor and individual spherical grain sizes were measured using ImageJ software^1^. Spheres were measured because they are a typical morphotype for magnetite and/or maghemite, which are among the most common transformation products of dissimilatory iron reduction. A minimum of 100, and up to 600, individual mineral grains across 10–20 frames were measured to ensure statistical significance.

A subset of samples was chosen for SAED and high-resolution TEM analyses at the UMass Electron Microscopy Center using a JEOL-JEM-2200FS transmission electron microscope operated at 200 kV and with a Schottky thermal field emitter source. The TEM is equipped with a TVIPS TemCam F416 for image acquisition and effective pixel size was calibrated using a gold nanoparticles standard. In addition to any “heat+cell” and “heat only” reacted samples, synthetic and pure nanophase mineral standards, namely ferrihydrite, maghemite, and magnetite, were visualized for reference. All SAED images were processed and analyzed using the DiffTools package (Mitchell, 2008) in DigitalMicrograph^TM^ (GMS3) (Gatan, Inc.) and distinguished based on *d*-spacing measurements. *D*-spacings represent the distance between planes of atoms in a crystal and were used to identify the crystalline phases present in a sample. Each diffraction pattern was processed five times to account for technical error in measurements. Errors in *d-*spacing measurements were ±2%. High-resolution TEM images were also analyzed using GMS3 for any lattice fringes. Lattice fringes were used to identify the mineral phase present in the sample by determining the spacing between the planes. Spacing measurements for lattice fringes were performed by generating a cross-correlation image based on gray-scale intensity of a selected ordered crystal, plotting relative intensity versus distance for the image, and then taking the total distance across all complete cycles and dividing it by the number of cycles. At least 10 cycles were measured for each ordered crystal, and the process repeated three times to account for technical error in measurement. Errors for lattice fringe spacing measurements were ±4%.

## Results

### Growth Screening on Nanophase Fe(III) (Oxyhydr)oxides

The synthetic nanophase Fe(III) (oxyhydr)oxide mineral characteristics and the results of the growth screen are provided in **Table 1**. Both *P. delaneyi* and *P. islandicum* showed growth on at least one synthesis of each of the nanophase minerals. *P. delaneyi* and *P. islandicum* produced the most Fe(II) (25 and 6 mM, respectively) and grew to the highest cell concentrations (9.0 × 10^7^ cells ml^-1^ and 4.6 × 10^7^ cells ml^-1^, respectively) on ferrihydrite. Lepidocrocite (Lep100615 and Lep030415) and akaganéite (Ak102315) were the next most reducible minerals, producing 2–3 mM Fe(II), which correlated with growth up to 2 × 10^7^ cells ml^-1^, although among these three minerals, Lep030415 supported successful growth and reduction only with *P. islandicum.* The least Fe(II) (<1–2 mM) was produced when both organisms were provided maghemite (Magh061815), goethite (Goet012315 and Goet011515), and hematite (Hem100915 and Hem022015). While a minimal amount of Fe(II) (1 mM) was produced with maghemite (Magh061815), both organisms grew to final cell concentrations of 1–1.7 × 10^7^ cells ml^-1^. *P. islandicum* favored Goet012315 among the goethite minerals and produced 2 mM Fe(II). *P. delaneyi* was unable to use this mineral for growth. Both organisms were able to use Goet011515, but minimal reduction (<1 mM) was observed. Only Hem100915 of the two-hematite minerals (Hem100915 and Hem022015) showed growth (<1 × 10^7^ cells ml^-1^) with little Fe(II) reduction (<1 mM).

**Table 1:** Synthetic nanophase mineral characteristics and results of growth screen.

Fe(III) (oxyhydr)oxide					Fe(II) produced (mM)^b^	

Mineral	Synthesis	Formula	Grain shape	Width^a^ (nm)	*Pyrodictium delaneyi*	*Pyrobaculum islandicum*
Ferrihydrite	Fh	5Fe_2_O_3_∙9H_2_O	Sphere	3 ± 1	25	6
Akaganéite	Ak102315	β-FeOOH	Lathe	41 ± 17	2	2
Lepidocrocite	Lep100615	γ-FeOOH	Platelet	6 ± 2	2	2
Lepidocrocite	Lep030415	γ-FeOOH	Platelet	31 ± 12	NG^c^	3
Maghemite	Magh061815	γ-Fe_2_O_3_	Irregular	6 ± 2	1	1
Goethite	Goet012315	α-FeOOH	Needles	3 ± 1	NG	2
Goethite	Goet011515	α-FeOOH	Needles	7 ± 2	< 1	< 1
Hematite	Hem100915	α-Fe_2_O_3_	Sphere	6 ± 1	< 1	< 1
Hematite	Hem022015	α-Fe_2_O_3_	Rhombohedral	36 ± 10	NG	NG

*^*a*^Grain shape and width/diameter was determined using TEM micrographs and represents individual grains and not aggregates. *^*b*^*Maximum Fe(II) reflects uncorrected values and is only listed if growth was observed after adaptation based on three successive transfers on the mineral type. *^*c*^*NG, no growth.*

### Growth and Fe(II) Production Rates

**FIGURE 1:**
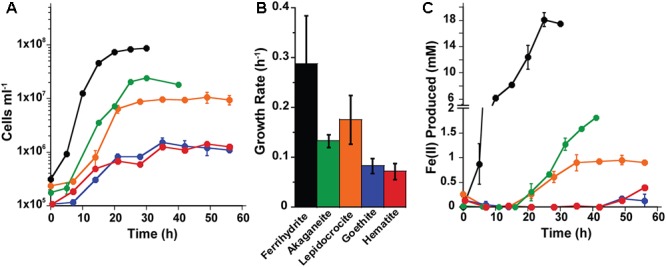
Growth **(A,B)** and Fe(II) production **(C)** kinetics for *Pyrodictium delaneyi* on synthetic nanophase Fe(III) (oxyhydr)oxides. *P. delaneyi* was grown autotrophically using 200 kPa H_2_:CO_2_ and synthetic nanophase Fe(III) (oxyhydr)oxides—ferrihydrite (black), akaganéite (green), lepidocrocite (orange), goethite (blue), and hematite (red) in a marine medium incubated at 90°C. Fe(II) concentrations represented are corrected for heat reacted growth medium (abiotic control): Fe(II) = Fe(II)_Heat+Cells_ – Fe(II)_Heat_. Fe(II) production appear delayed in the case of akaganéite and lepidocrocite despite growth due to this correction. Error bars represent the standard deviations **(A,C)** and 95% confidence intervals **(B)**.

Based on the growth screen, one mineral synthesis product for each type of Fe(III) (oxyhydr)oxide (e.g., Goet011515 or Goet012315) was chosen to measure growth and cell specific Fe(II) production rates in both organisms (Supplementary Table S1). The growth and iron reduction rates of *P. delaneyi* and *P. islandicum* differed based on the Fe(III) (oxyhydr)oxide used as a terminal electron acceptor. For *P. delaneyi*, growth rates varied between 0.29 ± 0.10 and 0.07 ± 0.02 h^-1^ (±95% confidence interval) depending upon the Fe(III) (oxyhydr)oxide used (**Figure 1**). *P. delaneyi* grew fastest on ferrihydrite, lepidocrocite, and akaganéite, with growth rates significantly different between ferrihydrite and akaganéite, but statistically significant differences were not seen between ferrihydrite and lepidocrocite or lepidocrocite and akaganéite growth rates (**Figure 1B** and Supplementary Table S1). Growth rates on hematite and goethite were significantly slower than those observed for ferrihydrite, lepidocrocite, and akaganéite, but not significantly different from each other (**Figure 1B**). The maximum cell concentrations for cultures grown on ferrihydrite were about an order of magnitude higher than on lepidocrocite and akaganéite, and nearly two orders of magnitude higher than on hematite and goethite (**Figure 1A**). Fe(II) concentrations increased in a cell-dependent manner for *P. delaneyi* when grown on ferrihydrite, lepidocrocite, and akaganéite, but not on hematite and goethite (**Figure 1C**). The maximum Fe(II) concentration for ferrihydrite was approximately 10–20 times higher than akaganéite or lepidocrocite, and approximately 50–100 times higher than hematite or goethite. There was no growth observed when *P. delaneyi* was grown solely on soluble iron such as Fe(III)-citrate, FeCl_3_, or FeCl_2_ (Supplementary Table S1), or in the absence of an electron acceptor. Additionally, when exponentially grown cells were heat-killed and exposed to ferrihydrite for up to 64 h, <1 mM Fe(II) was produced.

**FIGURE 2:**
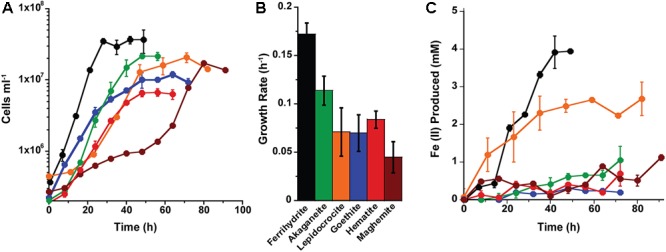
Growth **(A,B)** and Fe(II) production **(C)** kinetics for *Pyrobaculum islandicum* on synthetic nanophase Fe(III) (oxyhydr)oxides. *P. islandicum* was grown heterotrophically using 0.5% (wt vol^-1^) casein hydrolysate and synthetic nanophase Fe(III) (oxyhydr)oxides—ferrihydrite (black), akaganéite (green), lepidocrocite (orange), goethite (blue), hematite (red), and maghemite (brown) in a freshwater medium incubated at 95°C. Fe(II) concentrations represented are corrected for heat reacted growth medium (abiotic control): Fe(II) = Fe(II)_Heat+Cells_ – Fe(II)_Heat_. Error bars represent the standard deviations **(A,C)** and 95% confidence intervals **(B)**.

Growth rates for *P. islandicum* grown on the various nanophase Fe(III) (oxyhydr)oxides varied from 0.17 ± 0.01 to 0.05 ± 0.02 h^-1^ (±95% confidence interval) (**Figure 2**). Cells grew fastest, to the highest cell concentration, and produced the most Fe(II) (4 mM) with ferrihydrite (Supplementary Table S1). *P. islandicum* growth on akaganéite was slower than on ferrihydrite, but significantly faster than on lepidocrocite, goethite, hematite, and maghemite. The latter four minerals had the slowest growth rates for *P. islandicum* (**Figures 2A,B**). The maximum cell concentrations attained with akaganéite and lepidocrocite were approximately two times lower, while those reached with maghemite, goethite, and hematite were 3–7 times lower, when compared with ferrihydrite (**Figure 2A**). Similarly, the maximum Fe(II) concentration was approximately two times lower for lepidocrocite, three times lower for akaganéite, five times lower for hematite and maghemite, and 14 times lower for goethite when compared to ferrihydrite (**Figure 2C**). Unlike *P. delaneyi, P. islandicum* grew on Fe(III)-citrate and FeCl_3_ (Supplementary Table S1). It also grew on FeCl_2_, which was likely due to partial oxidation of the solution prior to growth. However, it did not grow fermentatively or in the absence of an electron acceptor. Growth on Fe(III)-citrate produced the most Fe(II) (10 mM) among all insoluble and soluble iron forms, but the growth rate was significantly slower than on ferrihydrite, but not significantly different from akaganéite, lepidocrocite, goethite, and hematite (Supplementary Table S1). Heat-killed cells when exposed to ferrihydrite for up to 64 h produced <1 mM Fe(II).

Because only a limited amount of Fe(II) was produced using goethite, hematite, and maghemite, Fe(II) production yields (fmol Fe^2+^cell^-1^) and cell-specific Fe(II) production rates (fmol Fe^2+^cell^-1^h^-1^) were only determined for ferrihydrite, lepidocrocite, and akaganéite grown cultures (Supplementary Table S1). For *P. delaneyi*, no significant differences were observed between the Fe(II) production yields for ferrihydrite and akaganéite, which were significantly higher than the yield for lepidocrocite. Despite the differences in yields, the cell-specific Fe(II) production rates for *P. delaneyi* grown on all three minerals were not significantly different. For *P. islandicum*, the Fe(II) production yield was highest and not significantly different between ferrihydrite and lepidocrocite. The yield for akaganéite was significantly lower than that for ferrihydrite but not statistically different from lepidocrocite. The cell specific Fe(II) production rate was highest for ferrihydrite, and followed by lepidocrocite and akaganéite, which were not significantly different from each other. Fe(III)-citrate had a similar cell-specific Fe(II) production rate to ferrihydrite, despite producing more Fe(II).

The reduction of ferrihydrite for both organisms occurred primarily as a solid phase transformation, with Fe(II) in the soluble phase representing 0.1 mM [∼3% of total Fe(II)] for *P. islandicum* and 1.6 mM [∼7.5% of total Fe(II)] for *P. delaneyi*. For akaganéite and lepidocrocite, significantly larger fractions of the total Fe(II) produced were present as soluble Fe(II). 0.6 mM [∼52% of total Fe(II)] and 1.1 mM [∼44% of total Fe(II)] were soluble when *P. islandicum* and *P. delaneyi*, respectively, were grown on akaganéite. Similarly, 0.8 mM [∼25% of total Fe(II)] and 1.0 mM [∼75% of total Fe(II)] were soluble when *P. islandicum* and *P. delaneyi*, respectively, were grown on lepidocrocite.

### Morphology and Phase of Mineral Transformations

The reduced mineral product formed during growth varied with mineral species used for growth and organism. Minerals produced by *P. delaneyi* were attracted to a magnet when grown on ferrihydrite and akaganéite but non-magnetic on lepidocrocite, hematite, and goethite. All the *P. delaneyi* mineral end-products were visually distinguishable from the abiotic heated control samples. In contrast, among the end-products of *P. islandicum* growth, only transformations with ferrihydrite, akaganéite, and lepidocrocite were visually distinguishable from the abiotic heated control samples. A black and magnetic product formed with ferrihydrite. All the other products were non-magnetic.

To further characterize the mineral transformations, TEM was used to size and quantify the transformed mineral end products. Only spherical mineral morphologies were sized because those are expected for magnetite, a previously identified mineral end-product for *P. delaneyi* and *P. islandicum* iron reduction (Kashefi and Lovley, 2000; Kashefi et al., 2008; Lin et al., 2014). Additionally, all biogenic mineral transformations (“heat+cells” condition) were directly compared to abiotic mineral transformations that might have resulted due to heat and the growth medium (“heat” condition). Growth medium with and without added cells and incubated at room temperature were also tested as abiotic controls, but they showed no morphological transformations from the initial minerals used in each experiment (data not shown).

**FIGURE 3:**
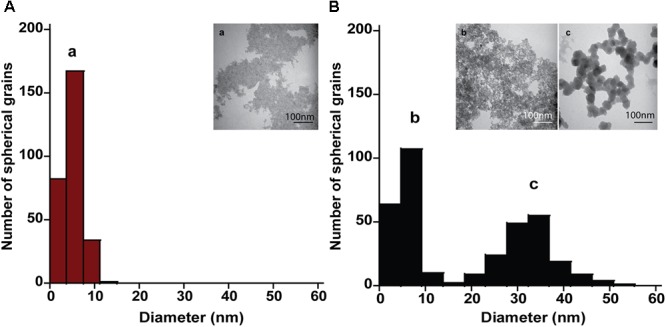
Size and morphology of ferrihydrite transformation products formed by *P. delaneyi.* Size distribution and electron micrographs of transformation products when ferrihydrite is incubated at 90°C in a marine medium without cells (“heat” abiotic condition) (**A**, a) and with exponentially grown *P. delaneyi* (“heat+cells” biotic condition) **(B**, b, c). Diameters for 100–600 individual particles were determined using electron micrographs. Representative grain sizes for the transformation products as well as corresponding electron micrographs are indicated (a–c).

**FIGURE 4:**
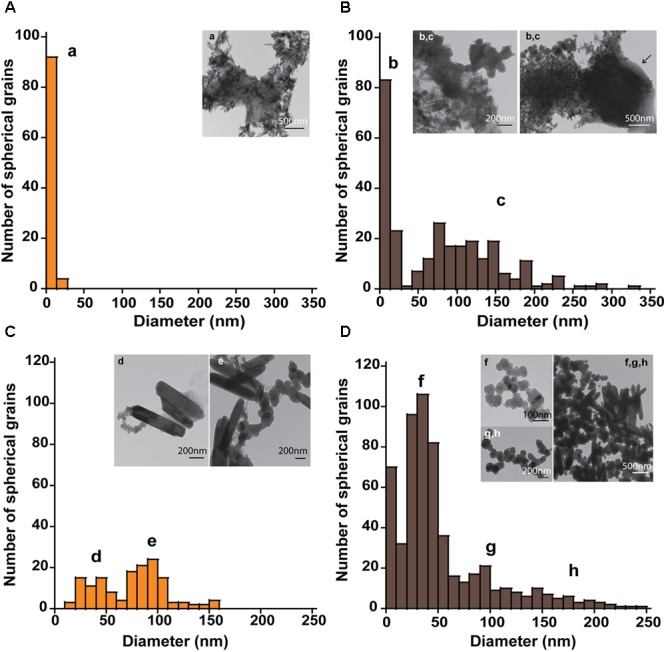
Size and morphology of lepidocrocite and akaganéite transformation products formed by *P. delaneyi.* Size distribution and electron micrographs of transformation products when lepidocrocite **(A,B)** and akaganéite **(C,D)** is incubated at 90°C in a marine medium without cells (“heat” abiotic condition) **(A,C)** and with exponentially grown *P. delaneyi* (“heat+cells” biotic condition) **(B,D)**. Diameters for 100–600 individual particles were determined using electron micrographs. Representative grain sizes for the transformation products as well as corresponding electron micrographs are indicated (a–h). Cells are indicated with an arrow for reference.

**FIGURE 5:**
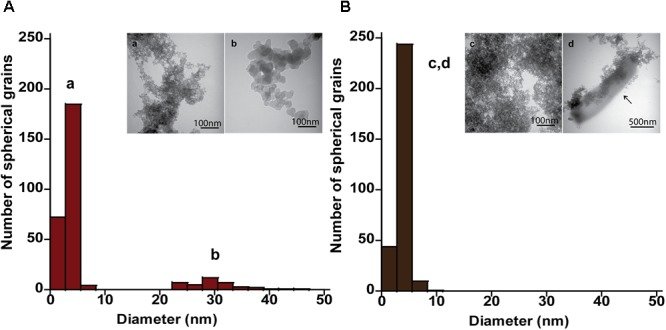
Size and morphology of ferrihydrite transformation products formed by *P. islandicum.* Size distribution and electron micrographs of transformation products when ferrihydrite is incubated at 95°C in a freshwater medium without cells (“heat” abiotic condition) (**A**, a, b) and with exponentially grown *P. islandicum* (“heat+cells” biotic condition) (**B**, c, d). Diameters for 100–600 individual particles were determined using electron micrographs. Representative grain sizes for the transformation products as well as corresponding electron micrographs are indicated (a–d). Cells are indicated with an arrow for reference.

The mineral end-products of bioreduction were morphologically distinguishable from the abiotic mineral end-products for *P. delaneyi* when grown on ferrihydrite (**Figure 3**), lepidocrocite (**Figures 4A,B**), and akaganéite (**Figures 4C,D**). *P. delaneyi* grown on ferrihydrite produced a spherical mineral grain that was larger (25–40 nm diameter) (**Figure 3B**) than the mineral product present in the abiotic control (**Figure 3A**). Spherical grains similar in size to ferrihydrite but less amorphous were also present among the bioreduced mineral population (**Figure 3B**). Similarly, with lepidocrocite, the bioreduced mineral produced by *P. delaneyi* comprised a population of larger spherical grains (50–300 nm diameter) (**Figure 4B**) compared to the abiotic control (<25 nm diameter) (**Figure 4A**). With akaganéite, a significantly wider range of larger grained spheres (10–250 nm diameter) (**Figure 4D**) was found in the bioreduced sample, and these were present at significantly higher abundance than in the abiotic control (**Figure 4C**). In contrast to *P. delaneyi, P. islandicum* produced a mineral end-product that was the same grain size as ferrihydrite (**Figure 5B**). A larger spherical mineral grain population (22–40 nm diameter) was found only in the abiotic control (**Figure 5A**). A similar larger spherical grain size was noted in the abiotic control of lepidocrocite and akaganéite (20–60 and 140–220 nm diameters, respectively) but not in the bioreduced end products for the organism (Supplementary Figure S2).

**FIGURE 6:**
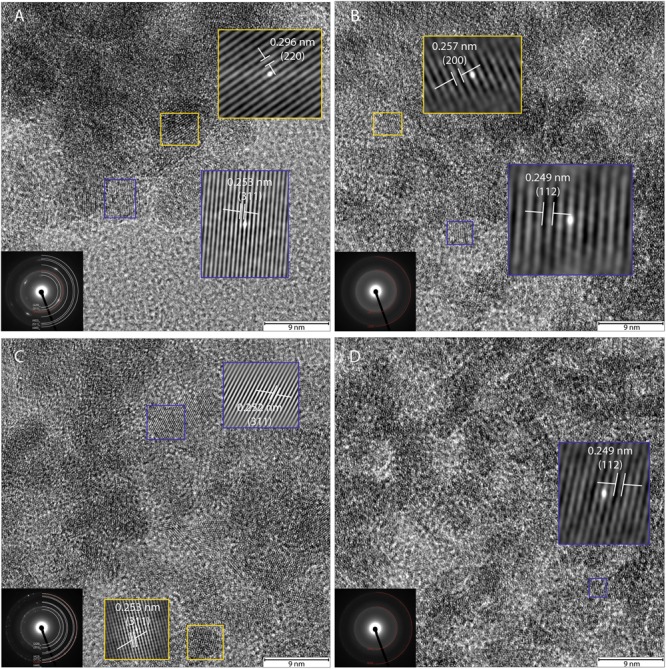
High-resolution TEM and SAED of transformation products of ferrihydrite incubations with exponentially grown *P. delaneyi* **(A)** and *P. islandicum* **(C)**. Marine medium without cells (“heat” abiotic condition) **(B)** and freshwater medium without cells (“heat” abiotic condition) **(D)** are also depicted for reference. For clarity, lattice fringes are magnified (not to scale) and shown using cross-correlation images based on gray-scale intensity of select ordered crystals. Insets (left corner) show the corresponding indexed SAED pattern for each condition. Each indexed ring as well as lattice fringe is denoted by its corresponding crystallographic plane (hkl). Subscript *f* means ferrihydrite, *m* means magnetite and maghemite.

High-resolution TEM images and SAED patterns of the “heat” and “heat+cells” experimental conditions confirmed changes in mineralogy and crystallinity of the bioreduced samples upon growth. High-resolution TEM images of ferrihydrite transformations by *P. delaneyi* and *P. islandicum* show mineral grains with higher structural order, as evidenced by lattice fringes, when compared to their respective abiotic controls (**Figure 6** and Supplementary Figure S3). Lattice fringes corresponding to the crystallographic planes (311), (220) and (111) (*d* = 0.253, 0.296, and 0.480 nm, respectively) [(111) data not shown], characteristic of magnetite (Haavik et al., 2000) were identified when *P. delaneyi* was grown with ferrihydrite (**Figure 6A**). This sample additionally contained lattice fringes corresponding to the crystallographic planes (200) and (112) (*d* = 0.257 and 0.249 nm) of ferrihydrite (Michel et al., 2007), the starting mineral (data not shown) (**Figure 6A**). High-resolution TEM images of *P. islandicum* grown on ferrihydrite suggest the formation of a magnetite and/or maghemite phase as indicated by lattice fringes for (311) (*d* = 0.253 and 0.252 nm) and (220) (*d* = 0.296 and 0.294 nm) planes [(220) data not shown] (Pecharromán et al., 1995; Haavik et al., 2000) (**Figure 6C**). Ferrihydrite lattice fringes for (200) (*d* = 0.257 nm) were also noted in this sample (**Figure 6C**). The abiotic controls for both organisms were characterized primarily by lattice fringes corresponding to (200) and (112) planes of the starting mineral ferrihydrite (**Figures 6B,D**).

Selected-area electron diffraction patterns of the “heat” condition for both organisms (**Figures 6B,D**, insets) showed characteristic diffuse rings that were indexed to the starting mineral ferrihydrite based on Michel et al. (2007). The bioreduced samples showed polycrystalline features, in the form of rings with discrete spots, which were indexed to magnetite and maghemite as well as the starting mineral ferrihydrite based on reference standards (Pecharromán et al., 1995; Haavik et al., 2000; Michel et al., 2007) (**Figures 6A,C**, insets).

## Discussion

This study demonstrates that a range of Fe(III) (oxyhydr)oxides of variable thermodynamic stability was bioreduced by two hyperthermophilic archaea when presented as nanophase minerals. It is the first study to examine the breadth and reduction susceptibility of Fe(III) (oxyhydr)oxides available as electron acceptors for hyperthermophilic archaea. These results illustrate that while many Fe(III) (oxyhydr)oxides are susceptible to reduction, the degree of conversion and reduction rate varies with Fe(III) (oxyhydr)oxide and microorganism. Ferrihydrite is the most bioavailable Fe(III) (oxyhydr)oxide for both *P. delaneyi* and *P. islandicum*, likely due to its poorly crystalline nature and low thermodynamic stability. Lepidocrocite and akaganéite are the next two most accessible mineral species for reduction by both organisms, as indicated by the maximum Fe(II) and cell concentrations attained. More thermodynamically stable phases such as hematite, goethite, and maghemite are poorly bioreduced in comparison. Crystal size changes of the transformed minerals were most distinguishable when *P. delaneyi* was grown with ferrihydrite, lepidocrocite, and akaganéite. Even among these minerals, lepidocrocite, and akaganéite showed the largest end-products, which may have implications for the longevity of these phases in nature, because larger grains are more resistant to phase changes with time.

A range of mineral characteristics constrain microbial Fe(III) (oxyhydr)oxide reduction, including surface area (Roden and Zachara, 1996; Roden, 2003, 2006), solubility (Bonneville et al., 2004, 2009), crystallinity and crystallite morphology (Cutting et al., 2009), size, and aggregation (Yan et al., 2008; Bose et al., 2009; Cutting et al., 2009; Bosch et al., 2010). These constraints can be understood by considering two interdependent factors—mineral surface energy and reactive surface site accessibility. Surface energy, as determined by crystal structure and crystallinity, dictates thermodynamic favorability of ligand- or microbe-mineral interaction. It is the basis of solubility and reactivity of mineral species, with ferrihydrite being the most soluble/reactive and hematite being the least soluble/reactive. However, when particles are very small (<60 nm equivalent spherical diameter), the trend between surface energy and mineral type can change. For instance, at <12 nm lepidocrocite can become thermodynamically more stable (less reactive) than hematite (Navrotsky et al., 2008). Additionally, morphology and crystallinity of a mineral can change overall reactivity. Not all surface sites are equal; accessibility to these reactive surface sites is necessary to facilitate dissolution or catalyze reduction.

In this study, monocrystalline size, mineral type, and morphology were varied. Consistent with previous findings for mesophilic bacteria *Geobacter* and *Shewanella*, no direct correlation was observed between mineral reactivity (bioavailability) and monocrystalline particle size when only nanoparticles were considered (Yan et al., 2008; Bose et al., 2009; Bosch et al., 2010). This is not unexpected for the minerals used in this study. While all the minerals were nanophase, few of them fell into the highly unique <12 nm size regime where reactivity trends can change between mineral species. However, increased reactivity and microbial reduction was seen in this study for *P. delaneyi* relative to previous incubations with macroaggregated lepidocrocite, goethite, hematite, and maghemite (Lin et al., 2014). This is consistent with the finding that nanoaggregates are more bioreactive than macroaggregates for a given mineral species (Bosch et al., 2010). It can also be attributed to the greater spatial bioaccessibility of nanophase minerals maintained as suspensions after synthesis. The results of this study also correlate well with the expected thermodynamic stability of the Fe(III) (oxyhydr)oxides. Growth and Fe(II) production were highest on less thermodynamically stable minerals such as ferrihydrite, lepidocrocite, and akaganéite relative to their more thermodynamically stable counterparts. In the case of *P. islandicum*, only minor differences in cell concentrations were observed regardless of mineral type despite different extents of Fe(II) production. The reducibility, accessibility, or reactivity of the mineral type may have influenced these differences. Variations in crystallite morphology also had an impact on the accessibility of an Fe(III) (oxyhydr)oxide. The two nanophase hematites (Hem100915 and Hem022015) showed distinct differences in grain morphology and size with Hem100915 occurring as spheres (6 nm) and Hem022015 as rhombohedra (36 nm). Only the smaller spheres of Hem100915 were amenable to growth and (minimal) reduction by both organisms. Although broad scale implications cannot be made from one example, morphology and shape of Fe(III) (oxyhydr)oxides deserve further attention regarding mineral constraints on microbial reduction among hyperthermophiles.

Physiological mechanisms may also be important in determining the extent and rates of Fe(III) (oxyhydr)oxide reduction. Due to the insoluble nature of Fe(III) (oxyhydr)oxides, mesophilic bacteria use a range of mechanisms to perform extracellular electron transfer. These include direct electron transfer using embedded enzymes in membranes or on extracellular appendages (e.g., pili or nanowires); reduction and release of extracellular electron shuttles for non-enzymatic, extracellular iron reduction; and secretion of iron chelators that solubilize iron and return it to the cell for reduction (Shi et al., 2016). *P. islandicum* has been shown to require direct contact with ferrihydrite for growth and mineral reduction and there is no evidence for the production of an electron shuttling compound (Feinberg et al., 2008). It is unknown if *P. delaneyi* requires direct contact with Fe(III) (oxyhydr)oxides for growth or if its growth and rate of iron reduction increase when an artificial electron shuttle is supplied. Additionally, little is known about variations in mechanism with different Fe(III) (oxyhydr)oxides. *P. delaneyi* grew to higher cell concentrations, produced more Fe(II), and generally exhibited higher rates of growth and Fe(II) production than *P. islandicum.* The presence of salts and organic compounds may also influence rates and extents of iron reduction by altering the reactivity of the mineral surfaces. While both microbes are crenarchaea, *P. delaneyi* is a marine hydrogenotroph from a hydrothermal vent that is unable to grow on soluble iron (Ver Eecke et al., 2009; Lin et al., 2014). In contrast, *P. islandicum* is a terrestrial facultative autotroph grown on peptides in this study that use both soluble iron and insoluble Fe(III) (oxyhydr)oxides for growth (Huber et al., 1987; Kashefi and Lovley, 2000). These differences likely influenced the rates and extent of reduction observed between the two organisms. For example, *P. islandicum* was able to reduce less ferrihydrite yet achieve a cell concentration similar to *P. delaneyi.*

The two organisms also differ in their number and type of predicted *c*-type cytochrome containing proteins, which are considered necessary for dissimilatory iron reduction in mesophilic bacteria (Shi et al., 2016) and in the hyperthermophilic euryarchaeon *Ferroglobus placidus* (Smith et al., 2015). The *P. delaneyi* genome encodes for 17 predicted *c*-type cytochrome proteins, eleven of which have multiple heme-binding motifs (Lin et al., 2016a) while the *P. islandicum* genome encodes only three predicted monoheme *c*-type cytochrome proteins (Feinberg et al., 2008). None of the *P. delaneyi c*-type cytochrome proteins is homologous to the monoheme cytochrome proteins found in *P. islandicum* or to any other *c*-type cytochrome proteins in other organisms outside of the *Pyrodictiaceae*. While the abundance of *c-*type cytochromes in mesophiles has been used as an indicator of respiratory versatility, the type (monoheme or polyheme) of *c*-type cytochrome protein has been linked to electron transfer efficiency (Mowat and Chapman, 2005; Shi et al., 2007). Therefore, differences in *c-*type cytochrome content in *P. delaneyi* and *P. islandicum* may contribute to rates of growth and iron reduction for these organisms. Furthermore, *F. placidus* expressed a different suite of *c*-type cytochrome proteins when grown on ferrihydrite relative to growth on soluble iron (Smith et al., 2015). Physiological status may be crucial in defining the rate and extent of reduction. In this study, *P. delaneyi* and *P. islandicum* were adapted to each Fe(III) (oxyhydr)oxide examined through at least three successive transfers prior to experimentation. This ensured evaluation of a physiological relevant process for each Fe(III) (oxyhydr)oxide.

The fate of Fe(II) produced and subsequent transformations vary with type, bioavailability, and rate and extent of reduction of an Fe(III) (oxyhydr)oxide. While ferrihydrite was the most successfully reduced mineral, the extent of Fe(II) produced and rates of reduction varied significantly for the two organisms. All other minerals show limited Fe(II) production in both organisms. This is reflected in colors of the transformed products, which only appear visibly darker for transformations with ferrihydrite, lepidocrocite, and akaganéite. The formation of magnetite, a common transformation and mineralization product for dissimilatory iron reduction, is primarily controlled by ferrous concentration and the supply rate (Zachara et al., 2002; Hansel et al., 2003). High Fe(II) concentrations and high supply rates result in Fe(II) sorption onto a mineral and subsequent nucleation of magnetite, but only above a threshold Fe(II) concentration (Zachara et al., 2002; Hansel et al., 2003). It is plausible that for the more crystalline and more thermodynamically stable minerals, magnetite formation is hindered because of the low Fe(II) concentrations and supply rates. Surface passivation of Fe(II) on the mineral surfaces or microbial cells may also prevent prolonged reduction (Roden and Urrutia, 1999, 2002; Roden et al., 2000). However, enhanced reduction for crystalline phases has been previously illustrated in the presence of various complexants and electron shuttling compounds (Urrutia et al., 1999; Roden and Urrutia, 2002; Cutting et al., 2009). Future studies will examine if the addition of chelators or electron shuttling compounds stimulates the rates and extent of reduction of more thermodynamically stable phases for the two organisms.

Another aspect of this study relates to the size and nature of the mineral transformations that occur, which depend both on the mineral used for growth and the microorganism. For *P. delaneyi*, there was an increase in particle size upon growth with ferrihydrite and lepidocrocite relative to heated abiotic controls. High-resolution TEM and SAED results demonstrated the formation of magnetite from ferrihydrite, which appeared to lie in the SPM size range (<30 nm diameter). These results were consistent with previous Mössbauer analyses that identified the *P. delaneyi* end-product as fine-grained (12 nm) magnetite (Lin et al., 2014). Increased grain size with bioreduction of Fe(III) (oxyhydr)oxides has previously been reported for *G. metallireducens* (Vali et al., 2004), *Shewanella* sp. PV-4 (Roh et al., 2006), and *Thermoanaerobacter* sp. TOR39 (Zhang et al., 1998; Li, 2012). The SD magnetite (30–100 nm) crystals formed in these studies had unique morphologies (e.g., hexagonal platelet, lath-like, tabular platelet, octahedral) and potential long-term stability relative to SPM phases (Zhang et al., 1998; Vali et al., 2004; Roh et al., 2006; Li, 2012). They are expected to persist longer, contribute to magnetization of natural sediments, and serve as potential biosignatures (Gibbs-Eggar et al., 1999; Li, 2012). While the exact mineral phase formed when *P. delaneyi* was grown with akaganéite and lepidocrocite is not known, if it is magnetite/maghemite, sizing results suggest that a fraction of it falls within the SD or multi-domain size ranges. These end-products may have greater stability than those formed by reduction of ferrihydrite. In contrast, *P. islandicum* reduction of ferrihydrite did not result in increased grain size despite producing a magnetite or maghemite end-product. The transformed minerals lie in the SPM size range and were smaller than those previously reported for *P. islandicum* grown autotrophically using H_2_:CO_2_ (Kashefi et al., 2008). Phase transformations for *P. islandicum* may have been limited by the presence of a phosphate buffer. Strong adsorption of phosphate to mineral surfaces is known to cause stabilization and prevent crystallization or nucleation of magnetite (Fredrickson et al., 1998).

## Conclusion

This study highlights the range of Fe(III) (oxyhydr)oxides of variable thermodynamic stabilities that can be used for growth by two hyperthermophilic archaea when presented as nanophase minerals. These findings open untapped culturing potential and new mineral biotransformations for life detection. Enrichments of bacteria using more thermodynamically stable oxides at mesophilic temperatures has resulted in the identification of novel Fe(III) reducers (Hori et al., 2010, 2015; Lentini et al., 2012), but similar efforts have not been made for hyperthermophiles. Future studies will examine the physiological mechanisms used by *P. delaneyi* and *P. islandicum* to transform different types and mixtures of synthetic nanophase Fe(III) (oxyhydr)oxides and identify the different mineral end-products generated by these hyperthermophiles and their long-term stability in nature.

## Author Contributions

SK, ES, and MD synthesized the minerals. SK and JH performed the growth and Fe(II) production kinetic experiments. SK performed the electron microscopy analyses. SK and JH wrote the paper with input from all the authors.

## Conflict of Interest Statement

The authors declare that the research was conducted in the absence of any commercial or financial relationships that could be construed as a potential conflict of interest.

## Footnotes

1 http://rsb.info.nih.gov/ij
