# Supplementary material for: Reduction and Morphological Transformation of Synthetic Nanophase Iron Oxide Minerals by Hyperthermophilic Archaea
Source: Front Microbiol. 2018 Jul 11;9:1550. doi: 10.3389/fmicb.2018.01550 (PMC6050373; doi:10.3389/fmicb.2018.01550)
Supplement: Supplementary file 1 [file Image_1.PDF]

Supplementary Material

Reduction and Morphological Transformation of Synthetic Nanophase Iron Oxide Minerals by Hyperthermophilic Archaea

Srishti Kashyap, Elizabeth C. Sklute, M. Darby Dyar, James F. Holden*

*** Correspondence:** Dr. James F. Holden: jholden@micro.umass.edu

This file contains:

Figures S1-S3

Table S1

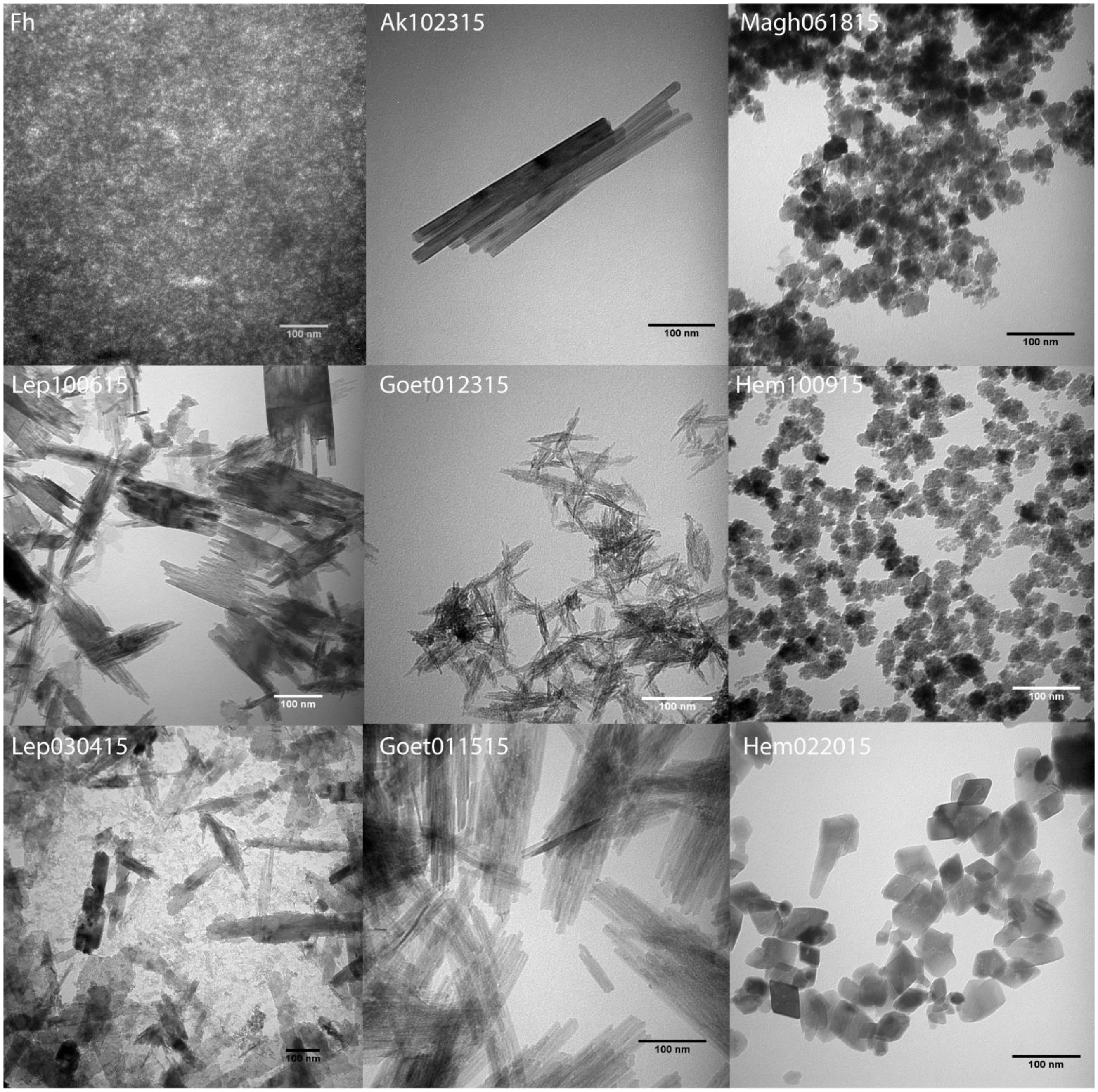

Supplementary Figure 1. Transmission electron micrographs of the synthetic Fe(III) (oxyhydr)oxide nanoparticles used for this study. *Fh*, ferrihydrite; *Ak102315,* akaganéite; *Magh061815*, maghemite; *Lep100615, Lep030415,* lepidocrocite; *Goet012315, Goet011515*, goethite; *Hem100915, Hem022015*, hematite. All scale bars are 100 nm (after Sklute et al., 2018).

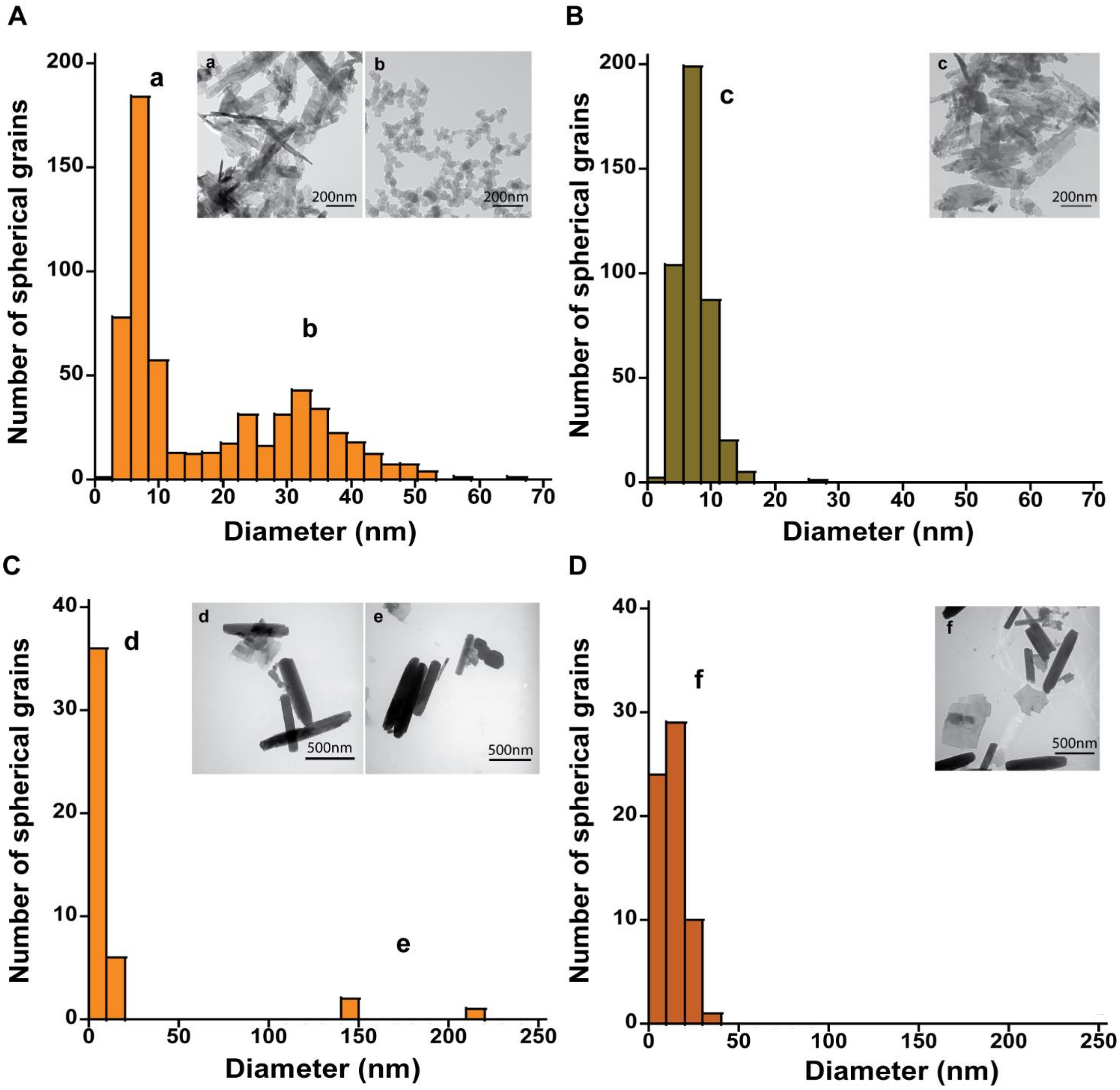

Supplementary Figure 2. Size and morphology of lepidocrocite and akaganéite transformation products formed by *P. islandicum*. Size distribution and electron micrographs of transformation products when lepidocrocite (A, B) and akaganéite (C, D) is incubated at 95°C in a freshwater medium without cells (‘heat’ abiotic condition) (A, C) and with exponentially grown *P. islandicum* (‘heat+cells’ biotic condition) (B, D). Diameters for 100-600 individual particles were determined using electron micrographs. Representative grain sizes for the transformation products as well as corresponding electron micrographs are indicated (a, b, c, d, e, f). Spherical grains are noted in all conditions, and a slight increase in grain size is noted only in the abiotic condition for both minerals but not for the biotic condition.

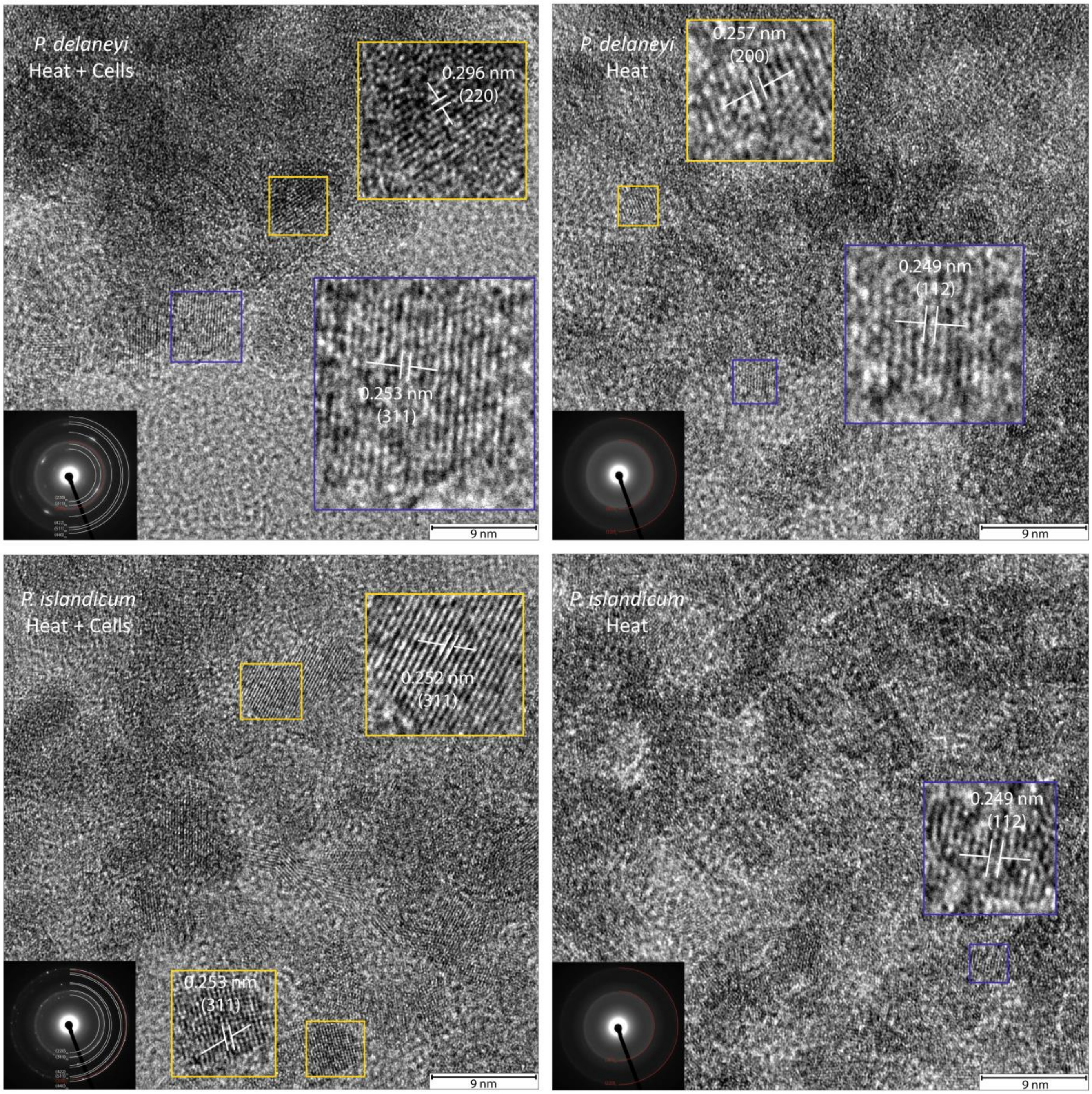

Supplementary Figure 3. High-resolution TEM and SAED of transformation products of ferrihydrite incubations with exponentially grown *P. delaneyi* and *P. islandicum* (left side). Marine medium without cells (*P. delaneyi* ‘heat’ abiotic condition) and freshwater medium without cells (*P. islandicum* ‘heat’ abiotic condition) are also depicted on the right side for reference. For clarity, lattice fringes are magnified (not to scale) to show select ordered crystals. Insets (left corner) show the corresponding indexed SAED pattern for each condition. Each indexed ring as well as lattice fringe is denoted by its corresponding crystallographic plane (hkl). Subscript *f* means ferrihydrite, *m* means magnetite and/or maghemite.

Table S1. Growth and Fe^{2+} production kinetics data for *P. delaneyi* and *P. islandicum*.

	Maximum Cell Concentration[a] ($\times 10^7$, ml^{-1})	Maximum Fe(II)[a,b] (mM)	Growth Rate[c] (k, h^{-1})	Yield[c] (Y) (fmol Fe^{2+} $cell^{-1}$)	Cell Specific Fe^{2+} Production Rate[c] (q) (fmol Fe^{2+} $cell^{-1}$ h^{-1})	Iron Reduction Rate[c] (μM h^{-1})
***Pyrodictium delaneyi*:**						
Ferrihydrite (Fh)	9.0 ± 0.3	19 ± 1.0	0.288 ± 0.096	170.0 ± 51.4	52.7 ± 25.6	812.8 ± 124.0
Akaganeite (Akag102315)	1.1 ± 0.03	1.8 ± 0.0	0.133 ± 0.013	180.0 ± 26.9	34.5 ± 6.2	58.4 ± 10.3
Lepidocrocite (Lep100615)	1.2 ± 0.3	1 ± 0.09	0.176 ± 0.049	75.3 ± 36.8	19.1 ± 10.8	18.1 ± 11.4
Goethite (Goet011515)[d]	0.2 ± 0.03	0.2 ± 0.03	0.083 ± 0.015	-	-	10.5 ± 5.1
Hematite (Hem100915)[d]	0.1 ± 0.003	0.4 ± 0.0	0.072 ± 0.016	-	-	4.4 ± 6.8
Maghemite (Magh061815)[e]	1.0 ± 0.2	0.7 ± 0.2	ND	ND	ND	ND
Fe^{3+}-Citrate	0	0	0	0	0	0
$FeCl_2$	0	0	0	0	0	0
$FeCl_3$	0	0	0	0	0	0
***Pyrobaculum islandicum*:**						
Ferrihydrite (Fh)	4.6 ± 0.4	4.2 ± 0.4	0.172 ± 0.012	85.2 ± 24.0	21.1 ± 6.1	96.9 ± 12.9
Akaganeite (Akag102315)	2.3 ± 0.2	1.3 ± 0.4	0.114 ± 0.015	21.0 ± 10.0	3.5 ± 1.7	16.7 ± 2.7
Lepidocrocite (Lep030415)	2.3 ± 0.3	2.4 ± 0.0	0.071 ± 0.025	51.4 ± 33.4	5.3 ± 3.9	15.7 ± 8.5
Goethite (Goet011515)[d]	1.3 ± 0.1	0.3 ± 0.03	0.070 ± 0.019	-	-	5.1 ± 2.2
Hematite (Hem100915)[d]	0.7 ± 0.08	0.9 ± 0.3	0.084 ± 0.009	-	-	4.0 ± 4.7
Maghemite (Magh061815)[d]	1.7 ± 0.1	0.9 ± 0.07	0.045 ± 0.016	-	-	7.4 ± 6.3
Fe^{3+}-Citrate[f]	6.5 ± 1.6	10.2 ± 0.3	0.111 ± 0.025	128.0 ± 25.2	20.5 ± 6.1	229.3 ± 42.5
$FeCl_2$[g]	0.8 ± 0.1	0.1 ± 0.1	0.087 ± 0.022	1.5 ± 2.1	0.2 ± 0.3	0.1 ± 0.3
$FeCl_3$[g]	1.9 ± 0.1	0.4 ± 0.03	0.104 ± 0.015	12.3 ± 4.8	1.85 ± 0.7	3.9 ± 1.5

[a] *± represents range of duplicate incubations.*
[b]*Fe(II) maximum values are corrected for heat reacted growth medium (abiotic control) values:* $Fe(II) = Fe(II)_{Heat+Cells} - Fe(II)_{Heat}$
[c] *± 95% confidence intervals.*
[d]*Cell and Fe(II) concentrations were too small to determine yield and cell specific* Fe^{2+} *production rates.*
[e]*ND, not determined due to limited maghemite synthesis product.*
[f]*Final concentration of 20 mM.*
[g]*Final concentration of 1.3mM.*
